# Effect of the Biopolymer Carrier on *Staphylococcus aureus* Bacteriophage Lytic Activity

**DOI:** 10.3390/biom12121875

**Published:** 2022-12-14

**Authors:** Liga Stipniece, Dace Rezevska, Juta Kroica, Karlis Racenis

**Affiliations:** 1 Rudolfs Cimdins Riga Biomaterials Innovations and Development Centre, Riga Technical University, LV-1007 Riga, Latvia; 2 Baltic Biomaterials Centre of Excellence, Headquarters at Riga Technical University, LV-1048 Riga, Latvia; 3 Department of Biology and Microbiology, Riga Stradins University, LV-1048 Riga, Latvia; 4 Joint Laboratory, Pauls Stradins Clinical University Hospital, LV-1002 Riga, Latvia; 5 Centre of Nephrology, Pauls Stradins Clinical University Hospital, LV-1002 Riga, Latvia

**Keywords:** biomaterials-associated infections, antimicrobials, bacteriophages, biopolymers

## Abstract

The use of implant materials is always associated with the risk of infection. Moreover, the effectiveness of antibiotics is reduced due to antibiotic-resistant pathogens. Thus, selecting the appropriate alternative antimicrobials for local delivery systems is correlated with successful infection management. We evaluated immobilization of the *S. aureus* specific bacteriophages in clinically recognized biopolymers, i.e., chitosan and alginate, to control the release profile of the antimicrobials. The high-titre *S. aureus* specific bacteriophages were prepared from commercial bacteriophage cocktails. The polymer mixtures with the propagated bacteriophages were then prepared. The stability of the *S. aureus* bacteriophages in the biopolymer solutions was assessed. In the case of chitosan, no plaques indicating the presence of the lytic bacteriophages were observed. The titre reduction of the *S. aureus* bacteriophages in the Na-alginate was below 1 log unit. Furthermore, the bacteriophages retained their lytic activity in the alginate after crosslinking with Ca^2+^ ions. The release of the lytic *S. aureus* bacteriophages from the Ca-alginate matrices in the TRIS-HCl buffer solution (pH 7.4 ± 0.2) was determined. After 72 h—0.292 ± 0.021% of bacteriophages from the Ca-alginate matrices were released. Thus, sustained release of the lytic *S. aureus* bacteriophages can be ensured.

## 1. Introduction

Biomaterials play a crucial role in modern medicine. The goal of using a variety of biomaterials is to restore or maintain the function and shape of a specific part of the human body. However, biomaterials-associated infections (BAIs) are one of the leading causes of implant failure. Moreover, biomaterials commonly serve as a colonization surface for bacteria to form a biofilm. Thus, the formation of a biofilm plays a pivotal role in the etiology of BAIs [1]. As a result, a complete removal of the implant, complex wound care, and prolonged courses of antibiotics are often required. In orthopaedics, BAIs occur in 2–5% of cases [2,3]. For the treatment of infections, antibiotics, such as tetracycline and vancomycin, are conventionally used in clinics [4]. This aims to reduce the potential for opportunistic bacteria to initiate infections and to reduce implant contamination risks during surgery or the postoperative period. Systemic administration of antibiotics has several disadvantages, including the development of dysbacteriosis. Thus, local delivery systems of the antibiotics have shown the following advantages over systemic approaches: (i) delivery close to the infection site, (ii) necessity of lower doses, and (iii) decreased risk of systemic toxicity and side effects [5]. Several approaches have been proposed to impart antibacterial properties to biomaterials effectively. The most popular methods are (i) entrapment of the antimicrobials in the pores of the implants, (ii) application of the antimicrobials as a part of the implants, or (iii) immobilization of the antimicrobials on the surface of the implants [6,7,8]. However, the effectiveness of antibiotics is reduced due to antibiotic-resistant pathogens; that is, some bacteria become resistant, or immune, to antibiotics. Antibiotic resistance is associated with increased morbidity, mortality, and treatment costs, and is considered a significant threat to human health worldwide [9]. Thus, selecting the appropriate antimicrobials and delivery systems/biomaterials is directly correlated with successful infection management. One of the alternatives to antibiotics are bacteriophages (phages). In the state of biofilm, the development of multidrug-resistant bacterial strains is significantly facilitated, and applying the anti-biofilm effect of bacteriophages in treating BAIs is genuinely promising. So far, we have successfully administered phage therapy for a patient with multidrug-resistant *Pseudomonas aeruginosa* osteomyelitis after a femoral fracture [10].

Bacteriophages are viruses that infect bacteria and use bacterial cells as hosts for their replication. Several characteristics of bacteriophages, including their ability to self-replicate and self-limit in their hosts, and their high specificity to species of bacteria, make them attractive candidates as therapeutic tools [11,12,13]. Bacteriophages are functionally divided into two groups: lysogenic and lytic. Lysogenic bacteriophages infect bacteria and live in them without any disturbance to the bacterial cell. Lytic bacteriophages are viruses that infect and kill bacteria. They do not infect human cells [14,15]. The outer surface of bacteriophages is made of proteins, making them sensitive to factors that denature proteins and impact viruses (e.g., exposure to organic solvents, elevated temperatures, high or low pH, ionic strength, etc.) [16]. The immobilization of bacteriophages by entrapment within matrices protects them against harsh conditions. However, the matrix used for bacteriophage immobilization could hinder contact of the immobilized bacteriophages with host bacteria in the surrounding medium, thus decreasing efficiency. Moreover, the concentration of the bacteriophages in the local environment must be sufficient to trigger the bacterial lysis. Therefore, the effective delivery of the bacteriophages involves maintaining lytic activity and tailoring a release rate. Clinically, the administration of the bacteriophages is usually performed by intravenous administration of high doses (≥10^6^ PFU/mL) during the first 6 h after surgery [17]. However, BAIs typically occur within 1 to 4 months after surgical transplantation. Thus, the short-term effect is insufficient to combat infections [18]. Delivery methods can be engineered to release bacteriophages in a burst for short-term effect, or a low, steady level for long-term effect.

According to the literature, most studies related to combining bacteriophages with materials, namely encapsulation, immobilization, etc., have been carried out in the food industry [19,20,21]. Analogous studies for medical applications are described relatively rarely. Due to their excellent properties, such as biodegradability, biopolymers are unique materials for medical applications, including in drug delivery systems [22,23]. Biodegradable polymers are considered one of the options for the long-term controlled delivery of antimicrobials, including bacteriophages, at the infection site. In this study, we evaluated the potential for immobilization of the bacteriophages in clinically recognized biopolymers, namely chitosan and alginate. Chitosan offers several advantages as it is naturally occurring, biodegradable, biocompatible, and non-toxic to mammalian cells [24]. However, chitosan is soluble only in dilute acidic solutions, which restricts its applications [25]. Alginate is a biocompatible, linear anionic polysaccharide derived from seaweed [26]. So far, both polymers have been successfully used in wound healing, tissue engineering, and drug delivery systems, making them excellent candidates for the local, controlled delivery of bacteriophages to the site of infection [27]. Most studies using alginate-based materials have mainly focused on incorporating drugs or antimicrobials into alginate without a chemical reaction. Common to all studies is the production of microcapsules by forming complexes between anionic alginate and cationic polysaccharides with characteristic antimicrobial properties (for example, chitosan) [28]. In a recent review article by Kim et al. (2021), current advances in the development of phage-delivering hydrogels, including alginates, for orthopedic implant-related bone infections, catheter-related urinary tract infections, and trauma-related wound infections have been reviewed [29].

*Staphylococcus aureus* (*S. aureus*) is one of the leading causes of skin, soft tissue, bone, and joint infections [30,31]. *S. aureus* has become the primary pathogen of increased importance due to its antibiotic resistance. Although drugs such as trimethoprim-sulfamethoxazole and vancomycin are still highly active against most *S. aureus* strains, resistance to vancomycin has already been reported, raising concerns about the possible reduction or loss of activity of this antibiotic soon [32]. Due to the prevalence of *S. aureus* infections worldwide, bacteriophages with anti-staphylococcal activity have received increased attention and awareness. Moreover, several reports on these bacteriophages have been published, including their genome analysis, host range traits, and activity [33,34,35,36].

Bourdin et al. (2014) have reported that pathogen coverage is dependent on the geographical area and epidemiological context and might reduce the effectiveness of commercially available phage cocktails [37]. Thus, flexible and locally adapted bacteriophages are required. Although the incorporation of bacteriophages into biopolymers has been reported previously, each newly prepared bacteriophage requires careful consideration to ensure that the delivery system’s chemistry and processing conditions are suited for the specific bacteriophage. The overall goal is to make antibacterial biomaterials that can retain their antibacterial activity within an identified period of time. The study aimed to evaluate stability in the biopolymers (chitosan or alginate) and the release of two propagated commercial bacteriophage cocktails against *S. aureus*.

## 2. Materials and Methods

### 2.1. Sample Preparation

#### 2.1.1. Preparation of the Bacteriophage Solutions

Two commercially available therapeutic bacteriophage cocktails, namely the Pyo Bacteriophage cocktail (hereinafter the corresponding bacteriophages are also called Pyo bacteriophages) and the Staphylococcal Bacteriophage cocktail (hereinafter the corresponding bacteriophages are also called Staph bacteriophages), produced by Eliava BioPreparations Ltd. (Tbilisi, Georgia) were used. According to the manufacturer’s information, the Pyo Bacteriophage cocktail leads to specific lysis of bacteria belonging to *Staphylococcus* (*S. aureus*), *Streptococcus* (*S. pyogenes*, *S. sanguis*, *S. salivarius*, *S. agalactiae*), *E. coli* (several types), *P. aeruginosa*, and *Proteus* (*P. mirabilis* and *P. vulgaris*) species. The Pyo Bacteriophage cocktail is used for the treatment and prophylaxis of purulent-inflammatory and enteric infectious diseases caused by the above-listed microorganisms. In turn, the Staphylococcal Bacteriophage cocktail leads to specific lysis of bacteria belonging to the *S. aureus* species. The Staphylococcal Bacteriophage cocktail is used in the treatment and prophylaxis of staphylococcal infections.

The procedure of preparing bacteriophage solutions before incorporating them into biopolymers included the following stages:

1. Culturing of the host bacterial strain. A solid agar plate was inoculated with the host strain (reference strain *S. aureus* (ATCC 25923)) and incubated overnight at 37 °C. Furthermore, the 3–5 bacterial colonies grown on the plate were inoculated in a liquid medium (trypticase soy broth (TSB, Oxoid, Basingstoke, Hampshire, UK)) and incubated overnight at 37 ± 1 °C.
2. Performing the plaque assay to determine the concentration of the bacteriophage cocktails. In test tubes, the overnight-grown host strain was mixed with serially diluted Staphylococcal and Pyo Bacteriophage cocktails. Then, 5 mL of molten 0.7% trypticase soy agar (TSA, Oxoid, Basingstoke, Hampshire, UK) was transferred to each test tube, and the obtained mixture was poured onto a solid TSA plate. After the top agar was solidified, the plates were inverted and placed in the incubator overnight at 37 ± 1 °C. The number of plaque-forming units per mL (PFU/mL) was counted.
3. Propagating commercial bacteriophage stocks to obtain higher titre bacteriophage lysates. After plaque assay testing, the webbed plates were selected and flooded with 5–7 mL of TSB. The supernatant and soft-overlay agar was selected. Afterwards, a 2% chloroform (CHCl_3_) treatment for 2 h at 4 °C, the removal of bacterial debris using centrifugation at 6000× *g* for 15 min at 4 °C, and filtration using a 0.20 μm filter were performed. The supernatant was then collected, and the final concentration (titre in terms of plaque-forming units per millilitre (PFU/mL)) was determined using plaque assay according to Equation (1):
  (1) $$\mathrm{Titer}=\;\frac{\mathrm{number}\;\mathrm{of}\;\mathrm{plaques}}{\mathrm{dilution}\;\mathrm{factor}\;\times \;\mathrm{volume}\;\mathrm{of}\;\mathrm{diluted}\;\mathrm{bacteriophage}\;\mathrm{in}\;\mathrm{mL}}$$

Stages 2 and 3 were repeated until the appropriate titre of the *S. aureus* bacteriophages was reached.

#### 2.1.2. Preparation of the Mixtures of Biopolymers/Bacteriophages

To prepare the 0.5%(*wt*/*vol*) chitosan solution, a commercial chitosan (coarse ground flakes, >75% deacetylated, 310–375 kDa, Sigma-Aldrich, Taufkirchen, Germany) was dissolved in a 1.0%(*vol*/*vol*) acetic acid (99.8–100.5%, Sigma-Aldrich) aqueous solution at ambient temperature.

To prepare the 1.0%(*wt*/*vol*) Na-alginate solution, a commercial Na-alginate (powder from brown algae, BioReagent, Merck, Darmstadt, Germany) was dissolved in deionized H_2_O under continuous stirring at ambient temperature overnight.

Before adding the bacteriophage solutions, the prepared 0.5%(*wt*/*vol*) chitosan and 1.0%(*wt*/*vol*) Na-alginate solutions were steam-sterilized in an autoclave Elara 11 (Tuttnauer, Breda, Noord-Brabant, The Netherlands) at 121 °C for 20 min (no drying). The propagated lytic bacteriophage cocktails were mixed with the sterilized biopolymers solutions at a volume ratio of 1:1.

For physicochemical characterization, the biopolymers and the biopolymers/bacteriophages films were prepared by the solution casting procedure. A fixed volume of the solutions (in case of the Na-alginate—2.5 mL, and in case of the chitosan—5 mL) were transferred into the weighing “boats” (made of polystyrene, chemically inert with a thermal resistance of up to 93 °C and capacity of 7 mL) and stored at 40 ± 1 °C until the solvent completely evaporated (up to 48 h). The films were then removed from the weighing “boats”.

Preparation of the Ca-alginate hydrogel beads (gel-cast green body with a diameter of 3–4 mm, without/with bacteriophages) involved crosslinking of the Na-alginate solution (without/with bacteriophages) with the CaCl_2_ (96%, Acros Organics, Geel, Belgium) solution. The Ca-alginate spheres were obtained by positioning the end of the syringe, with a needle diameter of 1.07 mm, within 90° and around 5 cm above the surface of the 250 mM CaCl_2_ solution and adding the Na-alginate solution dropwise to the CaCl_2_ solution under mild stirring. The resulting alginate gel droplets were stirred for an additional 30 min, then strained to remove the “excess” CaCl_2_ solution.

### 2.2. Characterization

The molecular structure of the chitosan and Na-alginate films and the Ca-alginate hydrogel dried beads (with a diameter of 1.5–1.8 mm) (with/without bacteriophages) was analysed using a Fourier-transform infrared spectrometer (FT-IR, Varian 800 Scimitar Series, Palo Alto, CA, USA). FT-IR spectra were recorded in the range of 400–4000 cm^−1^ with a spectral resolution of 4 cm^−1^ using an attenuated total reflectance module (ATR, GladiATR^TM^, Pike technologies, Fitchburg, MA, USA); 50 scans were averaged. Before every measurement, a background spectrum was taken and deducted from the sample spectrum.

The titre (PFU/mL) of the *S. aureus* bacteriophages in the solutions was determined by a plaque assay. A bacterial suspension was prepared from the *S. aureus* reference strain ATCC 25923 according to the McFarland standard with a turbidity of 0.5. The bacteriophage solutions (50 µL) were placed in a tube with 100 µL of the prepared bacterial suspension, and 5 mL TSB was added. After 24 h of incubation in a thermostat (37 ± 1 °C, 170 rpm), the contents (after dilution with TSB medium) of each tube underwent the plaque assay (as described in the Section 2.1.1). Plates containing 10–100 plaques were considered for further titre calculation according to Equation (1). The results are presented as the mean (of three compositions) encapsulation efficiency ± standard deviations (SD).

To determine the number of *S. aureus* bacteriophages embedded in the Ca-alginate hydrogels, the hydrogels were dissolved in a 0.1 M citrate buffer solution (pH 6.2) containing 0.09 M sodium citrate dihydrate (≥99%, food grade, Merck, Darmstadt, Germany) and 0.01 M citric acid (99 %, Merck, Darmstadt, Germany). To the samples, 500 μL of TSB was added and vortexed until a homogenous solution was obtained. Then, the plaque assay was executed to determine the titre of bacteriophages. Serial dilutions of the bacteria and the sample mixtures were made. Acquired mixtures were transferred onto TSA plates. After an overnight incubation period at 37 ± 1 °C, plates containing 10–100 plaques were considered for further titre calculation according to Equation (1). The encapsulation efficiency of the bacteriophages in the Ca-alginate was calculated according to Equation (2):

(2) $$\mathrm{Encapsulation}\;\mathrm{efficiency}=\frac{\mathrm{quantity}\;\mathrm{of}\;\mathrm{the}\;\mathrm{released}\;\mathrm{bacteriophages}\;}{\mathrm{quantity}\;\mathrm{of}\;\mathrm{the}\;\mathrm{added}\;\mathrm{bacteriophages}}\times 100$$

The results are presented as the mean (of three compositions) encapsulation efficiency ± standard deviations (SD).

To determine the stability of the Ca-alginate beads and the release of lytic *S. aureus* bacteriophages, the Ca-alginate samples were incubated in a tris(hydroxymethyl)aminomethane-hydrochloric acid (TRIS-HCl) buffer solution. The TRIS-HCl solution was prepared according to the EN ISO 10,993–14:2001, by dissolving 13.25 g tris(hydroxymethyl)aminomethane (TRIS, GR for analysis buffer substance, ACS, Reag.Ph.Eur, Merck, Darmstadt, Germany) in 500 mL deionized water under agitation and buffered at pH 7.4 ± 0.2 at 36.5 ± 0.5 °C with 1 M HCl (37%, ACS, ISO, Reag, Ph.Eur, Merck, Darmstadt, Germany), and filled up to 1 L. Samples were placed in microcentrifuge tubes and poured over with TRIS-HCl pre-heated to 37 °C (1 mL per 0.5 mg sample). The microcentrifuge tubes containing TRIS-HCl and the samples were incubated in the table-top environmental shaker-incubator at 37 ± 1 °C providing mild orbital shaking at 100 rpm. To evaluate the stability of the hydrogels, the incubated Ca-alginate beads, after specific time points (1 h, 6 h, 24 h, and 72 h), were strained and dried (40 °C, 24 h). Mean mass changes as well as standard deviations (SD) were calculated from three independent measurements. To determine bacteriophage release, the release medium (TRIS-HCl) was refreshed after 1, 6, 24, and 72 h. Three independent samples were analyzed at each time point. The titre of bacteriophages in the TRIS-HCl after incubation of the Ca-alginate hydrogels was determined using the plaque assay. Serial dilutions of the bacteria and the sample mixtures were made. Acquired mixtures with added molten 0.7% TSA were transferred onto TSA plates. After an overnight incubation at 37 ± 1 °C, plates containing 10–100 plaques were considered for further titre calculation in PFU/mL. The titre was calculated according to Equation (1). The results are presented as the mean (of three compositions) encapsulation efficiency ± standard deviations (SD).

## 3. Results and Discussion

### 3.1. Bacteriophages Propagation

The propagation procedure was crucial in increasing the original titre of the commercial bacteriophage cocktails. In the case of the Pyo Bacteriophage cocktail, the titre of the *S. aureus* bacteriophages increased from 1.2 × 10^6^ PFU/mL to 3.1 × 10^9^ PFU/mL, whereas in the case of the Staph Bacteriophage cocktail—from 5.6 × 10^5^ PFU/mL up to 1.0 × 10^9^ PFU/mL. The obtained high-titre bacteriophage solutions were further incorporated into the biopolymer matrices. Before preparing each new series of samples, the bacteriophage titre of the stock solution was re-determined, and the new value was used for the relevant calculations.

### 3.2. Molecular Structure of the Biopolymers

**Figure 1:**
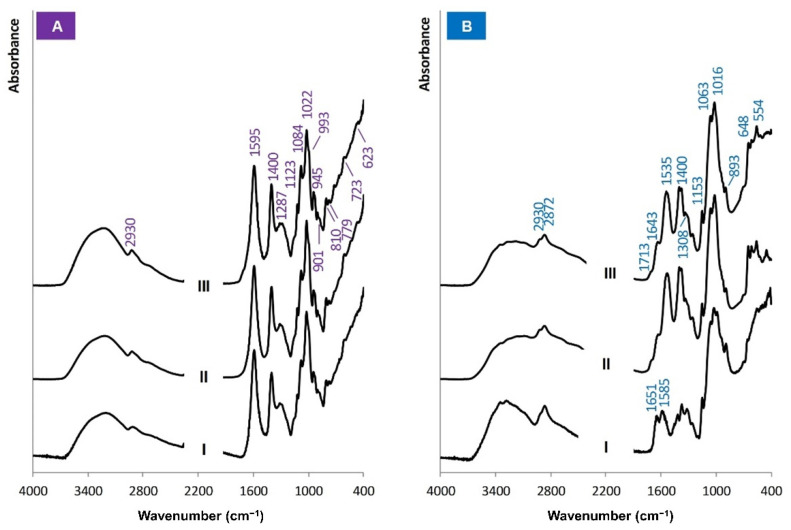
FT-IR spectra of biopolymers: (**A**-I) Na-alginate (commercial), Na-alginate film prepared from the solution (**A**-II) before and (**A**-III) after autoclaving, (**B**-I) chitosan (commercial), chitosan film prepared from the solution (**B**-II) before and (**B**-III) after autoclaving.

The molecular structure of the original materials (commercial Na-alginate and chitosan) and biopolymers films was analysed using FT-IR. From FT-IR spectra (Figure 1), it was concluded whether the molecular structure of the biopolymers was changed during the film preparation. Sterilization of the biopolymers solutions before the addition of the bacteriophages and production of the films was chosen considering that any sterilization method is undesirable for bacteriophages since they are microorganisms, the killing of which is the primary task of any sterilization method. Thus, it was crucial to understand whether sterilization in saturated water vapour (i.e., autoclaving) is suitable for processing the biopolymers solutions and whether such an approach does not change the molecular structure of the biopolymers.

In the FT-IR spectra of the Na-alginate (commercial biopolymer (Figure 1A-I)), film prepared by solution casting (Figure 1A-II), and film prepared from the autoclaved biopolymer’s solution (Figure 1A-III) characteristic absorbance bands of O-H, C-H, C=O, and C-O were detected. The O-H absorbance bands at 3000–3600 cm^−1^ are wide and extended, indicating a non-linear, branched structure. The C-H bond gives a weak signal at 2930 cm^−1^. All three spectra have two intensive absorbance bands with maxima at 1595 cm^−1^ and 1400 cm^−1^ corresponding to C=O and C-O bonds, respectively [38]. All spectra coincide, indicating that the molecular structure of the biopolymer has not changed during film preparation and autoclaving. Thus, the sterilization technique, namely autoclaving of the Na-alginate solution before the addition of bacteriophages, is suitable.

In the case of commercial chitosan (Figure 1B-I), a broad absorbance band in the region of 3000–3600 cm^−1^ characteristic to O-H bonds and low-intensity bands at 2930 and 2872 cm^−1^ characteristic to C-H bonds were detected in the FT-IR spectra. Stretching vibrations of C=O bonds at 1651 cm^−1^ and bending vibrations of N-H bonds at 1585 cm^−1^ represent amide groups and indicate partially acetylated chitosan [38]. Since chitosan is a water-insoluble biopolymer, it was dissolved in an acidic medium provided by the addition of acetic acid. This affected the appearance of amide groups’ characteristic absorbance bands (Figure 1B-II,III). The absorbance bands of the amide groups appeared as a singlet at 1535 cm^−1^. In the case of the raw material, the absorbance bands of the amide groups are a doublet (Figure 1B-I). The appearance of the characteristic absorbance bands of O-H (3000–3600 cm^−1^) and C-H (2930, 2852 cm^−1^) bonds also changed; their intensity decreased. However, the characteristic FT-IR absorbance bands of the chitosan films, prepared from the biopolymer solution before and after the autoclaving, match. It can be concluded that the molecular structure of the chitosan is slightly changed during the preparation of the solution. However, autoclaving does not further change the molecular structure of the chitosan. Thus, the chosen sterilization method of chitosan, namely autoclaving the biopolymer’s solution at 121 °C for 20 min, is considered suitable.

**Figure 2:**
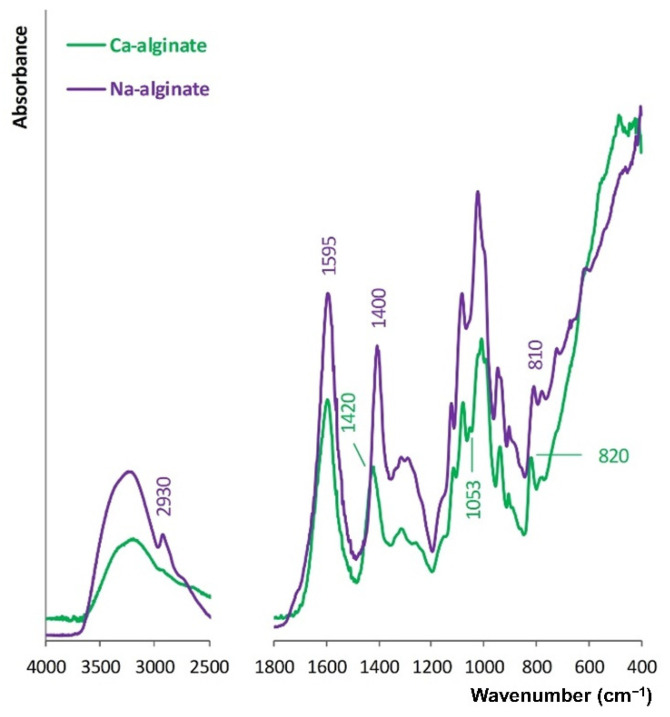
FT-IR spectra of the Na-alginate and the Ca-alginate.

Na-alginate was cross-linked with Ca^2+^ ions. FT-IR spectra of the Na-alginate and Ca-alginate films prepared from the autoclaved biopolymer solutions are compared in Figure 2.

Absorbance bands attributed to stretching vibrations of O-H bonds for Ca-alginate are lower than for Na-alginate. This is due to the role of O-H and -COO- groups in the formation of a chelating structure with Ca^2+^ ions. Accordingly, hydrogen bonding between O-H functional groups decreases. Moreover, the cations’ charge density, radius, and atomic weight change when Ca^2+^ ions replace Na^+^ ions. Hence, this shifting of the C-O absorbance bands from 1400 to 1420 cm^−1^ occurs, C-O stretching vibration absorption increases, and an absorbance band at 1053 cm^−1^ appears [39]. Na-alginate has an absorption band at 2930 cm^−1^ that is assigned to the stretching vibration of C-H in the six-membered ring, which cannot be observed in the spectrum of Ca-alginate. Crosslinking with Ca^2+^ ions promoted the formation of the egg-box model. Thus, the stretching vibration of C-H in the six-membered ring of the Ca-alginate molecule is limited [40].

**Figure 3:**
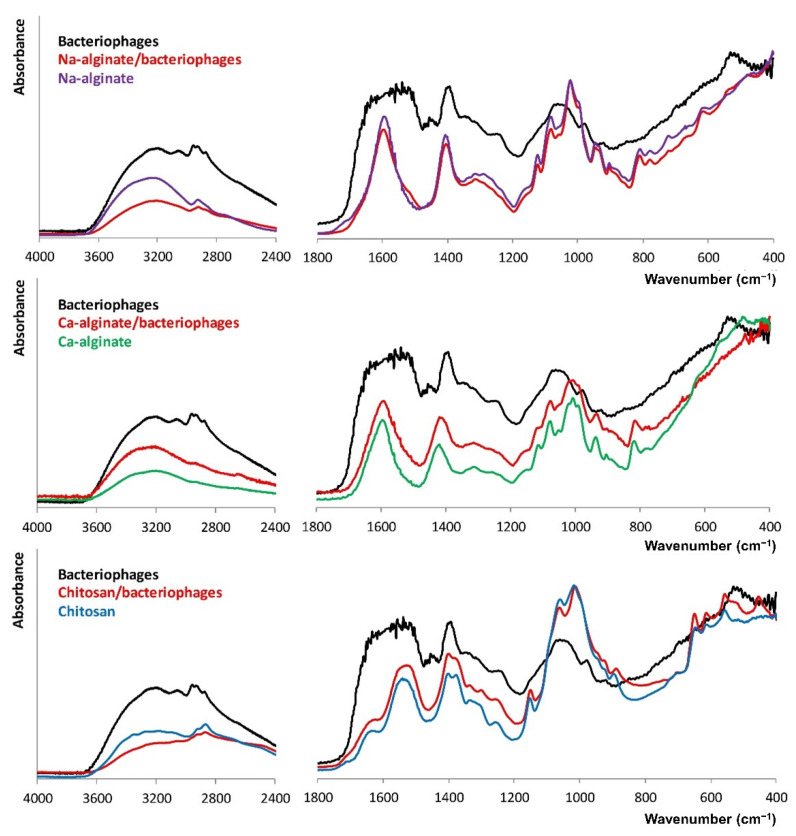
FT-IR spectra of the Pyo bacteriophages containing biopolymer films compared to the FT-IR spectra of the plain biopolymer films and dried bacteriophage suspensions.

The molecular structure of biopolymers films after the addition of bacteriophage suspensions was characterized and is shown in Figure 3. For both kinds of bacteriophages, FT-IR bands between 1800 and 900 cm^−1^ correlate with the presence of proteins, polysaccharides, and nucleic acid macromolecules. The range 1200–900 cm^−1^ is typical for carbohydrates, 1500–1200 cm^−1^ corresponds to the carboxyl groups, and 1700–1500 cm^−1^ is characteristic of proteins, and within the 3000–2800 cm^−1^, the lipid fraction is observed [41]. The main difference between the FT-IR spectra of the biopolymers films and the bacteriophages containing biopolymers films was a slight increase in intensity or broadening caused by the overlapping of the FT-IR bands.

### 3.3. Bacteriophages Stability

The potential of using the chosen biopolymers (Na-alginate and chitosan) for immobilizing commercial bacteriophage stocks was investigated. First, the interaction of bacteriophages and biopolymer solutions was analysed.

**Figure 4:**
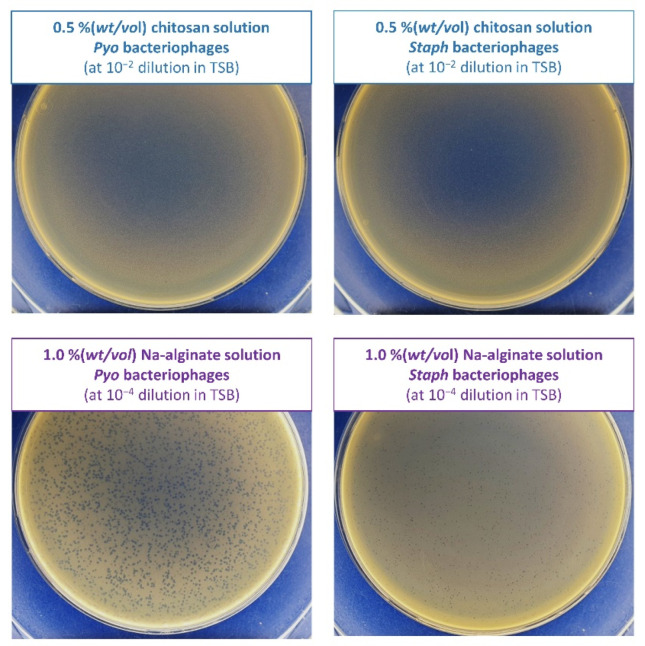
Plaques formed in the agar overlay by the bacteriophages in the biopolymer solutions.

Qualitatively, the lytic activity was assessed by plaque formation in a lawn of *S. aureus* bacteria due to lysis by bacteriophages (Figure 4). In the case of the chitosan solution, no plaques (clean areas typical for lytic bacteriophage) indicating the presence of viable bacteriophages were observed. Thus, adding the bacteriophages to the 0.5%(*wt*/*vol*) chitosan resulted in a complete loss of titre. This is due to the solution’s acidic nature (pH 4.07). Given this observation, chitosan was not used in further studies, and the emphasis was placed on the effect of Na-alginate on the viability of bacteriophages within commercial stocks. In the case of the Na-alginate solution, bacteriophages retained their lytic activity, which was confirmed by the formed plaques. In the case of the Staph bacteriophage, the plaques were smaller. The size of the plaque is proportional to the efficiency of adsorption, the length of the latent period, and the burst size of the bacteriophage. Also, the bacteriophages with larger heads (a significant fraction of myophages) tend to form smaller plaques compared to the bacteriophages with smaller heads (a significant fraction of sipho- and podophages). This is because larger virions (with larger heads) would diffuse more slowly through the top agar layer than smaller ones, thus resulting in smaller plaques [42].

Quantitatively, the number of bacteriophages in the Na-alginate solution was compared with the expected number, and a reduction of titre was calculated in each case (Table 1).

Both commercial bacteriophage stocks showed good stability in the Na-alginate solution with the titre reduction of less than 1 log unit. For further studies, the bacteriophage showing higher stability, i.e., with smaller titre reduction, was chosen to sustain high concentration. Thus, further studies were performed using the Pyo bacteriophages. In addition, as shown in Figure 4, they formed larger and, therefore, easier to see and quantify, which is essential from a practical point of view.

The viability of the bacteriophages during drying (at 40 °C) of the Na-alginate was determined, and the results are summarized in Table 2. The results confirmed that increasing the drying time at 40 °C to 24 h showed insignificant titre reduction until no plaques were formed in the completely dried Na-alginate samples (144 h).

Since the drying of Na-alginate for biomaterials application is required, this approach has no potential for the development of bacteriophage delivery systems. Therefore, Ca-alginate hydrogel for embedding the lytic bacteriophage commercial cocktails were evaluated. Ca-alginate hydrogel was disrupted, and the titre of the immobilized bacteriophages was determined. All Ca-alginate hydrogel samples contained bacteriophages showing lytic activity, as evidenced by plaques formed in the agar overlay. The titre reduction after crosslinking the Na-alginate with CaCl_2_, i.e., the incorporation of the bacteriophages into the Ca-alginate hydrogel, was below 1 log unit (Table 3). In addition, the titre of the bacteriophages embedded in Ca-alginate hydrogel remained close to the planned or nominal value.

To compare, Cobb et al. (2019) reported on Ca-alginate injectable hydrogels for the delivery of genetically modified phage vB_EfaS_LM99 (against *S. aureus*), reaching an embedded phage concentration of 3 × 10^7^ PFU/mL [43]. In another study, Ma et al. (2012) embedded *S. aureus* phage K in Ca-alginate microspheres for oral delivery, achieving a bacteriophage encapsulation efficiency of 95% of added 10^8^ PFU/mL [44]. The results of these studies, as well as ours, confirm that ionic crosslinking of Na-alginate solution containing bacteriophages is a suitable method to efficiently embed *S. aureus* bacteriophages into Ca-alginate hydrogels.

### 3.4. Ca-Alginate Stability and Bacteriophages Release

**Figure 5:**
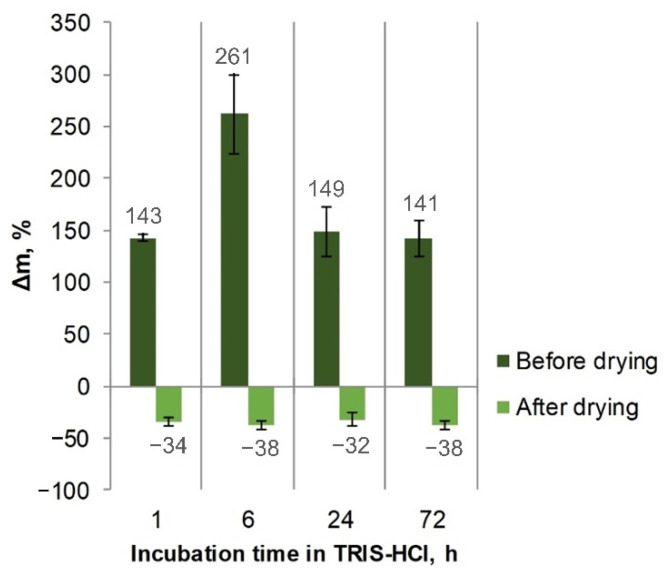
Mass changes of Ca-alginate hydrogel beads after incubation in TRIS-HCl buffer.

After incubation of the Ca-alginate microspheres in the TRIS-HCl buffer, a liquid uptake was observed. The highest fluid uptake was after 6 h. After 24 and 72 h, the mass gain was lower, which could be attributed to the fact that degradation of the hydrogel had occurred. After drying the incubated samples, weight loss was observed, i.e., the samples were partially dissolved (Figure 5).

**Figure 6:**
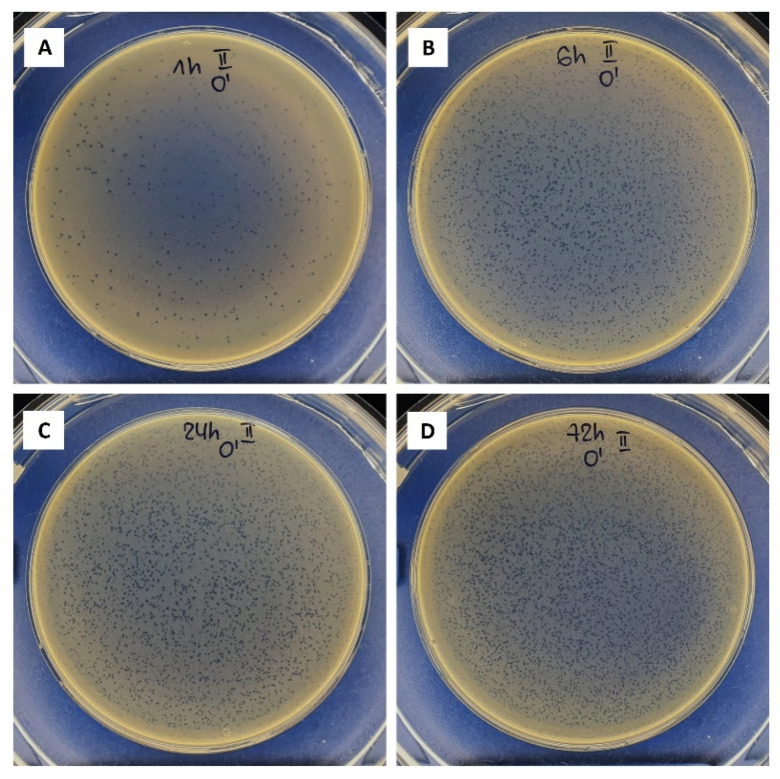
Plaques formed by the Pyo bacteriophages released from Ca-alginate hydrogel in the TRIS-HCl buffer after incubation for (**A**)—1 h, (**B**)—6 h, (**C**)—24 h, and (**D**)—72 h (no dilution).

Thus, it is concluded that the bacteriophage delivery occurs through the swelling–disintegration–degradation process of the alginate structure. This is in agreement with Barros et al.’s (2020) observations described in [45]. The bacteriophage stability in the TRIS-HCl buffer was assessed. Lytic bacteriophage stocks were stable in this medium as no titre reduction was observed. Figure 6 shows the plaques formed by the Pyo bacteriophages when being released from the Ca-alginate hydrogel in the TRIS-HCl buffer after various time periods.

**Figure 7:**
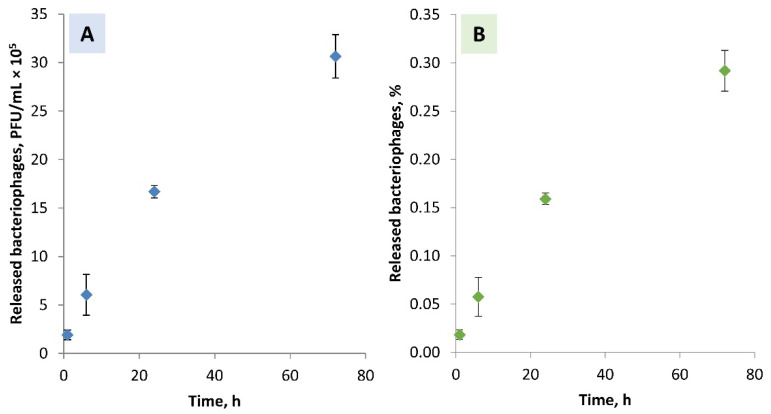
The cumulative amount of the Pyo bacteriophages released from the Ca-alginate hydrogel in the TRIS-HCl buffer ((**A**)—PFU/mL and (**B**)—% from the determined amount of the bacteriophages ((2.1 ± 0.2) × 10^8^ PFU/mL) in the Ca-alginate hydrogel).

A gradual increase in the titre of released bacteriophages was observed for 72 h (Figure 7). After 1 h—0.018 ± 0.005%, 6 h—0.058 ± 0.020%, 24 h—0.159 ± 0.006%, and 72 h—0.292 ± 0.021% of bacteriophages immobilized in the Ca-alginate matrices were released. Naturally, as the difference in incubation time increases (from 1 to 6 h (5 h), from 6 to 24 h (18 h), and from 24 to 72 h (48 h)), the number of the released bacteriophages also increases in each period. This was also observed visually by the formed plaques (Figure 6). The number of bacteriophages released in the first 6 h reached 6 × 10^4^ PFU/mL. However, it is difficult to speculate whether such an amount would be therapeutically effective due to the lack of clinical studies on the local supply of bacteriophages using biomaterials. These results suggest that such an approach can ensure a sustained local supply of bacteriophages.

## 4. Conclusions

The high-titre *S. aureus* lytic bacteriophage suspensions were prepared from commercial bacteriophage cocktails (Staphylococcal Bacteriophage and Pyo Bacteriophage) using a propagation procedure. The acidic medium of the chitosan solution is lethal for the bacteriophages. Drying of the Na-alginate is critical for the loss of lytic activity of the bacteriophages. In turn, the bacteriophages retained their lytic activity in the Na-alginate solution, and the titre reduction of bacteriophages was below 1 log unit. Furthermore, the immobilized bacteriophages retained their lytic activity in the Ca-alginate hydrogel, which can serve as bacteriophage delivery systems for the sustained local delivery of antimicrobials. Further biological studies are needed to predict the necessary concentration of bacteriophages in this type of system to achieve a therapeutic effect.

## Figures and Tables

**Table 1:** Titre of the bacteriophages in 1.0%(*wt*/*vol*) Na-alginate solution.

	Pyo Bacteriophages		Staph Bacteriophages	
	Titre, PFU/mL	Titre Reduction, Log Units	Titre, PFU/mL	Titre Reduction, Log Units
Expected	1.6 × 10^9^	0.22 ± 0.01	5.0 × 10^8^	0.30 ± 0.02
Measured	(9.6 ± 0.3) × 10^8^		(2.5 ± 0.1) × 10^8^	

**Table 2:** Titre of the Pyo bacteriophages during drying of the Na-alginate.

	Stock Solution, PFU/mL	Titre after Drying at 40 °C, PFU/mL		
		5 h	24 h	144 h
Stock solution of Pyo bacteriophages	4.8 × 10^8^	N/D *	N/D	N/D
Pyo bacteriophages/ Na-alginate	(2.2 ± 0.7) × 10^8^	(1.2 ± 0.2) × 10^8^	(1.3 ± 0.2) × 10^8^	0

* N/D—not determined.

**Table 3:** Titre of the Pyo bacteriophages embedded in the Ca-alginate hydrogels.

	Titre, PFU/mL	Titre Reduction, Log Units	
Expected	2.4 × 10^8^	0.03 ± 0.01	0.05 ± 0.01
Na-alginate solution	(2.2 ± 0.7) × 10^8^		
		0.01 ± 0.01	
Ca-alginate hydrogel	(2.1 ± 0.2) × 10^8^		

## Data Availability

Not applicable.

## References

[1] Lu Y., Cai W.J., Ren Z., Han P. (2022). The Role of Staphylococcal Biofilm on the Surface of Implants in Orthopedic Infection. Microorganisms.

[2] Rosas S., Ong A.C., Buller L.T., Sabeh K.G., Law T.Y., Roche M.W., Hernandez V.H. (2017). Season of the year influences infection rates following total hip arthroplasty. World J. Orthop..

[3] Johnson C.T., Garcia A.J. (2015). Scaffold-based anti-infection strategies in bone repair. Ann. Biomed. Eng..

[4] Kim B.-N., Kim E.S., Oh M.-D. (2014). Oral antibiotic treatment of staphylococcal bone and joint infections in adults. J. Antimicrob. Chemother..

[5] Wassif R.K., Elkayal M., Shamma R.N., Elkheshen S.A. (2021). Recent advances in the local antibiotics delivery systems for management of osteomyelitis. Drug Deliv..

[6] Katsikogianni M., Missirlis Y.F. (2004). Concise review of mechanisms of bacterial adhesion to biomaterials and of techniques used in estimating bacteria-material interactions. Eur. Cells Mater..

[7] Campoccia D., Montanaro L., Renata C. (2006). The significance of infection related to orthopedic devices and issues of antibiotic resistance. Biomaterials.

[8] Romanò C.L., Scarponi S., Gallazzi E., Romanò D., Drago L. (2015). Antibacterial coating of implants in orthopaedics and trauma: A classification proposal in an evolving panorama. J. Orthop. Surg. Res..

[9] Blair J.M.A., Webber M.A., Baylay A.J., Ogbolu D.O., Piddock L.J.V. (2015). Molecular mechanisms of antibiotic resistance. Nat. Rev. Microbiol..

[10] Racenis K., Rezevska D., Madelane M., Lavrinovics E., Djebara S., Petersons A., Kroica J. (2022). Use of phage cocktail BFC 1.10 in combination with ceftazidime-avibactam in the treatment of multidrug-resistant *Pseudomonas aeruginosa* femur osteomyelitis—A case report. Front. Med..

[11] Qadir M.I., Mobeen T., Masood A. (2018). Phage therapy: Progress in pharmacokinetics. Braz. J. Pharm. Sci..

[12] Monk A.B., Rees C.D., Barrow P., Hagens S., Harper D.R. (2010). Bacteriophage applications: Where are we now?. Lett. Appl. Microbial..

[13] Thung T.Y., Lee E., Premarathne J.M.K.J.K., Nurzafirah M., Kuan C.H., Elexson N., Tan C.W., Malcolm T.T.H., New C.Y., Ramzi O.S.B. (2018). Bacteriophages and their applications. Food Res..

[14] Davies E.V., Winstanley C., Fothergill J.L., James C.E. (2016). The role of temperate bacteriophages in bacterial infection. FEMS Microbiol. Lett..

[15] Hyman P. (2019). Phages for phage therapy: Isolation, characterization, and host range breadth. Pharmaceuticals.

[16] Fulgione A., Ianniello F., Papaianni M., Contaldi F., Sgamma T., Giannini C., Pastore S., Velotta R., Della Ventura B., Roveri N. (2019). Biomimetic hydroxyapatite nanocrystals are an active carrier for Salmonella bacteriophages. Int. J. Nanomed..

[17] Vasilev K., Cook J., Griesser H.J. (2009). Antibacterial surfaces for biomedical devices. Expert Rev. Med. Devices.

[18] Zimmerli W. (2014). Clinical presentation and treatment of orthopaedic implant-associated infection. J. Intern. Med..

[19] Choinska-Pulit A., Mitula P., Sliwka P., Laba W., Skaradzinska A. (2015). Bacteriophage encapsulation: Trends and potential applications. Trends Food Sci. Technol..

[20] Lone A., Anany H., Hakeem M., Aguis L., Avdjian A.C., Bouget M., Atashi A., Brovko L., Rochefort D., Griffiths M.W. (2016). Development of prototypes of bioactive packaging materials based on immobilized bacteriophages for control of growth of bacterial pathogens in foods. Int. J. Food Microbiol..

[21] O’Connell L., Marcoux P.R., Roupioz Y. (2021). Strategies for Surface Immobilization of Whole Bacteriophages: A Review. ACS Biomater. Sci. Eng..

[22] Rebelo R., Fernandes M., Fangueiro R. (2017). Biopolymers in medical implants: A brief review. Procedia Eng..

[23] Stipniece L., Salma-Ancane K., Rjabovs V., Juhnevica I., Turks M., Narkevica I., Berzina-Cimdina L. (2016). Development of functionalized hydroxyapatite/poly(vinyl alcohol) composites. J. Cryst. Growth.

[24] Hosseinidoust Z., Olsson A.L.J., Tufenkji N. (2014). Going viral: Designing bioactive surfaces with bacteriophage. Colloids Surf. B Biointerfaces.

[25] Munoz-Bonilla A., Echeverria C., Sonseca A., Arrieta M.P., Fernandez-Garcia M. (2019). Bio-based polymers with antimicrobial properties towards sustainable development. Materials.

[26] Szekalska M., Pucilowska A., Szymanska E., Ciosek P., Winnicka K. (2016). Alginate: Current use and future perspectives in pharmaceutical and biomedical applications. Int. J. Polym. Sci..

[27] Rotman S.G., Sumrall E., Ziadlou R., Grijpma D.W., Richards R.G., Eglin D., Moriarty T.F. (2020). Local bacteriophage delivery for treatment and prevention of bacterial infections. Front. Microbiol..

[28] Wang Y.L., Hu J.J. (2021). Sub-100-micron calcium-alginate microspheres: Preparation by nitrogen flow focusing, dependence of spherical shape on gas streams and a drug carrier using acetaminophen as a model drug. Carbohydr. Polym..

[29] Kim H.Y., Chang R.Y.K., Morales S., Chan H.K. (2021). Bacteriophage-delivering hydrogels: Current progress in combating antibiotic resistant bacterial infection. Antibiotics.

[30] Arciola C.R., An Y.H., Campoccia D., Donati M.E., Montanaro L. (2005). Etiology of implant orthopedic infections: A survey on 1027 clinical isolates. Int. J. Artif. Organs.

[31] Kaplan S.L. (2014). Recent lessons for the management of bone and joint infections. J. Infect..

[32] Appelbaum P.C. (2006). The emergence of vancomycin-intermediate and vancomycin-resistant *Staphylococcus aureus*. Clin. Microbiol. Infect..

[33] Abatángelo V., Bacci N.P., Boncompain C.A., Amadio A.F., Carrasco S., Suarez C.A., Morbidoni H.R. (2017). Broad-range lytic bacteriophages that kill *Staphylococcus aureus* local field strains. PLoS ONE.

[34] Xia G., Wolz C. (2014). Phages of Staphylococcus aureus and their impact on host evolution. Infect. Genet. Evol..

[35] Azam A.H., Tanji Y. (2019). Peculiarities of Staphylococcus aureus phages and their possible application in phage therapy. Appl. Microb. Biotechnol..

[36] Fokine A., Rossmann M.G. (2014). Molecular architecture of tailed double-stranded DNA phages. Bacteriophage.

[37] Bourdin G., Navarro A., Sarker S.A., Pittet A.C., Qadri F., Sultana S., Cravioto A., Talukder K.A., Reuteler G., Brussow H. (2014). Coverage of diarrhoea-associated *Escherichia coli* isolates from different origins with two types of phage cocktails. Microb. Biotechnol..

[38] IR Spectrum Table & Chart. https://www.sigmaaldrich.com/LV/en/technical-documents/technical-article/analytical-chemistry/photometry-and-reflectometry/ir-spectrum-table.

[39] Peretz S., Florea-Spiroiu M., Anghel D.-F., Bala D., Stoian C., Zgherea G. (2013). Preparation of porous calcium alginate beads and their use for adsorption of O-nitrophenol from aqueous solutions. Microfluid Nanoeng..

[40] Pang Y., Xi F., Luo J., Liu G., Guo T., Zhang C. (2018). An alginate film-based degradable triboelectric nanogenerator. RSC Adv..

[41] Olszak T., Zarnowiec P., Kaca W., Danis-Wlodarczyk K., Augustyniak D., Drevinek P., de Soyza A., McClean S., Drulis-Kawa Z. (2015). In vitro and in vivo antibacterial activity of environmental bacteriophages against *Pseudomonas aeruginosa* strains from cystic fibrosis patients. Appl. Microbiol. Biotechnol..

[42] Jurczak-Kurek A., Gasior T., Nejman-Falenczyk B., Bloch S., Dydecka A., Topka G., Necel A., Jakubowska-Deredas M., Narajczyk M., Richert M. (2016). Biodiversity of bacteriophages: Morphological and biological properties of a large group of phages isolated from urban sewage. Sci. Rep..

[43] Cobb L.H., Park J., Swanson E.A., Beard M.C., McCabe E.M., Rourke A.S., Seo K.S., Olivier A.K., Priddy L.B. (2019). CRISPR-Cas9 modified bacteriophage for treatment of Staphylococcus aureus induced osteomyelitis and soft tissue infection. PLoS ONE.

[44] Ma Y., Pacan J.C., Wang Q., Sabour P.M., Huang X., Xu Y. (2012). Enhanced alginate microspheres as means of oral delivery of bacteriophage for reducing *Staphylococcus aureus* intestinal carriage. Food Hydrocoll..

[45] Barros J.A.R., Melo L.D.R., Silva R.A.R.D., Ferraz M.P., Azeredo J.C.V.R., Pinheiro V.M.C., Colaco B.J.A., Fernandes M.H.R., Gomes P.S., Monteiro F.J. (2020). Encapsulated bacteriophages in alginate-nanohydroxyapatite hydrogel as a novel delivery system to prevent orthopedic implant-associated infections. Nanomedicine.

